# Emergency Department Food Insecurity Screening, Food Voucher Distribution and Utilization: A Prospective Cohort Study

**DOI:** 10.5811/westjem.18513

**Published:** 2024-09-19

**Authors:** Alexander J. Ulintz, Seema S. Patel, Katherine Anderson, Kevin Walters, Tyler J. Stepsis, Michael S. Lyons, Peter S. Pang

**Affiliations:** * The Ohio State University College of Medicine, Department of Emergency Medicine, Columbus, Ohio; † Indiana University School of Medicine, Department of Emergency Medicine, Indianapolis, Indiana

## Abstract

**Objective:**

Food insecurity is a prevalent social risk among emergency department (ED) patients. Patients who may benefit from food insecurity resources may be identified via ED-based screening; however, many patients experience difficulty accessing resources after discharge. Co-locating resources in or near the ED may improve utilization by patients, but this approach remains largely unstudied. This study characterized the acceptance and use of a food voucher redeemable at a hospital food market for patients who screened positive for food insecurity during their ED visit.

**Methods:**

This prospective cohort study, conducted at a single county-funded ED, included consecutive adult patients who presented on weekdays between 8 am–8 pm from July–October 2022 and consented to research participation. We excluded patients who required resuscitation on arrival or could not provide written informed consent in English. Study participants completed a paper version of the two-question Hunger Vital Sign screening tool, administered by research staff. Participants who screened positive received a uniquely numbered $30 food voucher redeemable at the hospital’s co-located food market. Voucher redemption was quantified through regular evaluation of market receipt records at 30-day intervals. The primary outcome was the proportion of redeemed vouchers. Secondary outcomes included the proportion of participants screening positive for food insecurity, proportion of participants accepting vouchers, and associated descriptive statistics.

**Results:**

Of the 396 eligible individuals approached, 377 (95.2%) consented and completed food insecurity screening. Most were middle-aged (median 53 years, interquartile range 30–58 years), 191 were female (50.4%), 242 were Black (63.9%), and 343 were non-Hispanic (91.0%). Of the participants, 228 (60.2%) screened positive for food insecurity and 224 received vouchers (98.2%), of which 86 were redeemed (38.4%) a median of nine days after the ED visit.

**Conclusion:**

A high proportion of participants screened positive for food insecurity and accepted food vouchers; however, less than half of all vouchers were redeemed at the co-located food market. These results imply ED food voucher distribution for food insecurity is feasible, but co-location of resources alone may be insufficient in addressing the social risk and alludes to a limited understanding of facilitators and barriers to resource utilization following ED-based social needs screening.

Population Health Research Capsule What do we already know about this issue?

*Food insecurity is common among ED patients. Screening identifies individuals with this social risk, but little evidence guides referral.*

What was the research question?

*Will patients who screened positive for food insecurity in the ED accept a $30 food voucher redeemable at a co-located hospital market?*

What was the major finding of the study?

*98.2% of patients who screened positive for food insecurity accepted a voucher, but only 38.4% had redeemed them at a median of nine days later.*

How does this improve population health?

*Referral to co-located resources for food insecurity is feasible, but programs should consider accessibility and patient preferences in addressing social risks identified in the ED.*

## INTRODUCTION

Social risks, defined as adverse social conditions associated with poor health, are common among emergency department (ED) patients and influence their health outcomes.^1^ ^,^ ^2^ Food insecurity is one prevalent social risk among ED patients that is associated with progression and exacerbation of chronic disease, frequent acute care use, and increased all-cause mortality.^3^ ^–^ ^10^ Previous studies of ED-based food insecurity screening and referral to resources identified patients with this social risk; however, many patients who received resource referrals had difficulty connecting to these resources after discharge.^11^ ^,^ ^12^ Co-locating resources in or near the ED for patients who screen positive for food insecurity represents one potential solution that directly connects patients to targeted resources; however, little evidence supports this model.

Within the context of food insecurity resource co-location, case reports describe a “food bag program” piloted during the COVID-19 pandemic, which provided any discharged ED patient with a 1–2 day supply of shelf-stable food.^13^ However, this was primarily an operational project that prioritized food distribution and was limited in its conclusions regarding food insecurity screening and resource uptake due to co-location.^13^ Thus, it remains poorly understood whether co-locating resources in or near the ED to address this social risk lead to increased resource acceptance or utilization. In this study we sought to characterize the acceptance and use of a $30 food voucher redeemable at a hospital food market by study participants who screened positive for food insecurity during their ED visit.

## METHODS

### Study Design, Setting, and Participants

This prospective cohort study screened consecutive consenting patients presenting to the ED for food insecurity and provided a food voucher to participants who screened positive. This study was approved by the Indiana University School of Medicine Institutional Review Board (IRB #13829).The study site was a 95-room county-funded ED with over 100,000 annual visits. All patients arriving to the ED are first triaged in the waiting room or ambulance bay by a nurse; patients who do not require emergent stabilization based on appearance and chief complaint are evaluated in one of 24 intake rooms. Intake rooms are private rooms where a complete nursing evaluation and the first patient-physician interaction takes place.

This study included ED patients aged ≥18 years who presented on weekdays between 8 am–8 pm from July 1–October 31, 2022 and were evaluated in an intake room during their ED visit. The study protocol excluded patients who were minors, were placed in a non-intake room (due to requiring immediate resuscitation or a 1:1 nursing intervention), or who were unable to provide informed consent in English. Research assistants used the electronic health record to assess patients for eligibility upon their arrival in triage and approached eligible participants for consent once moved to a private intake room. Eligible participants were made aware of the purpose of the study and that their survey responses could make them eligible for a food voucher at the hospital food market.

### Screening Tool Distribution and Screening Data Collection

Consented participants received a paper version of the US Department of Agriculture binary question Hunger Vital Sign screening tool (“Within the past 12 months we worried whether our food would run out before we got money to buy more,” and “Within the past 12 months the food we bought just didn’t last and we didn’t have money to get more”).^14^ Participants used a provided writing instrument to check yes or no to each screening tool question. A paper version was chosen based on prior work by Gonzales et al (2021), which demonstrated that 75% of patients preferred food insecurity screening via paper as opposed to verbal responses; however, the consent process did notify participants that if they could not read or write, the research assistant (RA) could read the screening to questions to them.^15^ If the patient declined assistance in reading or filling out the screening tool, the RA left the room for 10 minutes and returned to collect the completed screening tool. The RA directly entered screening tool results into a predefined data collection instrument, REDCap. We collected and managed study data using REDCap electronic data capture tools hosted at Indiana University, which included the patient’s health record number for longitudinal tracking. Paper screening tools were then destroyed via the hospital’s confidential-document disposal system.

### Intervention

If the participant screened negative for food insecurity, no further intervention was performed. If food insecurity was identified on the screening tool, the participant received a $30 food voucher redeemable at the hospital’s co-located food market.^16^ The “Fresh for You” hospital-based market was designed by the health system to address food insecurity by providing patients, visitors, and staff easy access to fresh produce, prepared foods, healthy snacks, convenience ingredients, kitchen utensils, and pantry staples at affordable prices and in a convenient location.^17^ The food market is open weekdays from 10:30 am–6 pm and is located approximately 600 feet from the ED entrance, positioned near a bus stop and the parking garage. Prior to this study, a similar screening tool and voucher referral system had been used in select outpatient practices; the value of the voucher was chosen as it was similar to the outpatient practices. Each food voucher had a random three-digit code on the back, and this was recorded in the RedCap database prior to distribution by the research staff. In addition to the food voucher (study intervention), participants screening positive for food insecurity also received resources that were standard of care prior to this study, which included printed community resources for food insecurity. If a food voucher was redeemed, it was marked with the date and time of redemption by market staff. At 30-day intervals, the study team queried hospital food-market receipt records to determine whether a voucher had been redeemed and secondarily performed patient chart review for primary care follow-up visits.

### Outcomes

The primary outcome was the proportion of food vouchers redeemed by participants who screened positive for food insecurity. Secondary outcomes included the proportion of participants screening positive for food insecurity, proportion of individuals with outpatient follow-up after their ED visit, and demographic descriptions of these groups.

### Statistical Methods

The analysis plan included descriptive data analysis with frequencies, proportions, and medians with interquartile range (IQR). We did not calculate an a priori sample size due to enrollment being limited by the number of available vouchers. A post hoc power calculation for detecting differences between participants who redeemed or did not redeem a food voucher demonstrated less than 80% power; thus, statistical comparison was not performed, and only descriptive statistics are reported. All statistical analyses were performed using STATA IC version 17 (StataCorp, LLC; College Station, TX).

## RESULTS

**Figure 1.**
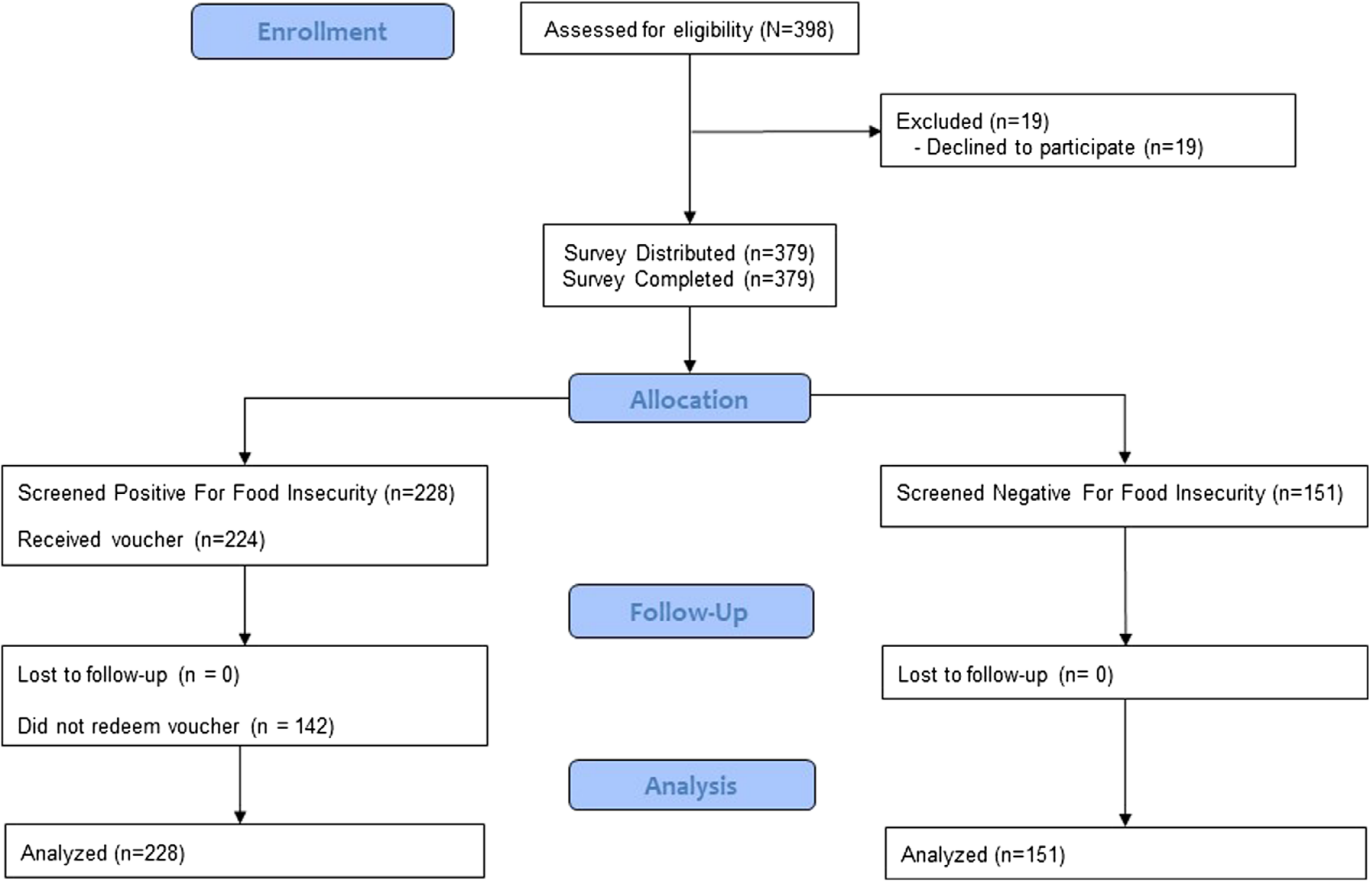
Study CONSORT diagram.

Research assistants approached 396 eligible individuals, of whom 379 consented and completed the food insecurity screening tool (Figure 1). No individual was screened more than once during the study period. Most participants were middle-aged (median 53 years, IQR 30–58 years); 191 identified as female (50.4%); 242 as Black (63.9%); and 343 as non-Hispanic, (91.0%); 234 reported having a primary care physician (61.7%). Most participants (228) screened positive for food insecurity (60.2%) (Table 1). Of these, 194 participants (51.2%) worried about food running out before having money to buy more, 207 respondents (54.6%) reported food not lasting long enough and not having money for more, and 175 respondents (46.2%) reported both concerns. (Table 2).

**Table 1.** Demographic description of enrolled participants, stratified by presence or absence of food insecurity.

Variable	All participants (N = 379)	Food insecurity present (n = 228)	Food insecurity absent (n = 151)
Gender, *n (column %)*			
Male	188 (49.6)	106 (46.5)	82 (54.3)
Female	191 (50.4)	122 (53.5)	69 (45.7)
Age, *median (IQR)*	53 (30–58)	45 (32–57)	40 (29–59)
Race, *n (column %)*			
Black	242 (63.9)	149 (65.4)	93 (61.6)
Native Hawaiian/Pacific Islander	1 (0.2)	1 (0.4)	0
White	133 (35.1)	78 (34.2)	55 (36.4)
Missing	3 (0.8)	0	3
Ethnicity, *n (column %)*			
Hispanic or Latino/a	34 (9.0)	20 (8.8)	14 (9.4)
Non-Hispanic	343 (91.0)	208 (91.2)	135 (90.6)
Missing	2	0	2
Access to care, *n (column %)*			
PCP prior to study	234 (61.7)	141 (62.4)	93 (61.6)

*IQR*, interquartile range; *PCP*, primary care physician.

**Table 2.** Hunger Vital Signs question responses and screening results (N = 379).

Question response	n (%)
*“Within the past 12 months we worried whether our food would run out before we got money to buy more”*	194 (51.2)
*“Within the past 12 months the food we bought just didn’t last and we didn’t have money to get more”*	207 (54.6)
Screening Result	
Screened positive for food insecurity	228 (60.2)
Answered yes to only one question	51 (22.4)
Answered yes to both questions	175 (76.8)
Screened negative for food insecurity	151 (39.8)

**Figure 2.**
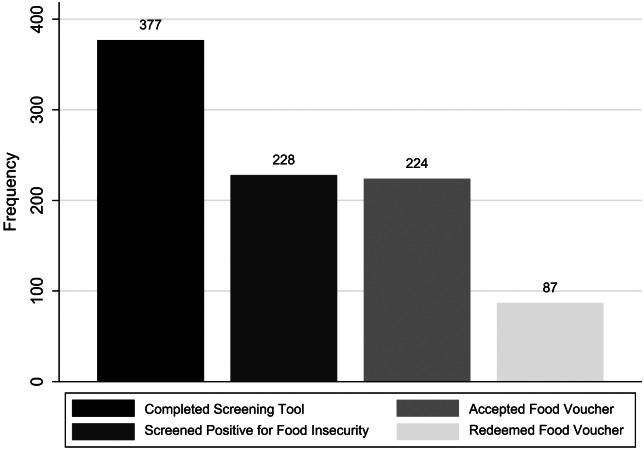
Frequency of screening tool completion, positive screening for food insecurity, voucher acceptance by patient, and voucher redemption.

The RAs distributed 224 vouchers (98.2% of participants who screened positive) and observed 86 (38.4%) redemptions within 30 days of distribution (Figure 2). The median time to voucher redemption was nine days (IQR 9–19 days). Of participants screening positive for food insecurity, 98 (43.0%) had primary care follow-up within 90 days of the ED visit. The median time to primary care follow-up was 41 days (IQR 21–67 days). Of note, 39 participants (17.1% of those who screened positive for food insecurity) neither redeemed a food voucher nor attended primary care follow-up (Table 3).

**Table 3.** Food insecurity after distribution, voucher redemption, and primary care physician follow-up (n = 228).

Variable	n (%)
Food voucher	
Vouchers distributed	224 (98.2)
Vouchers redeemed	86 (38.4)
Time-to-redemption, *median (IQR)*	9 days (9–19)
Follow-up	
PCP appointment within 90 days	98 (43.3)
Time-to-appointment, *median (IQR)*	41 days (21–67)
Food voucher and PCP follow up	
Both used	12 (5.3)
Neither used	39 (17.3)

*IQR*, interquartile range; *PCP*, primary care physician.

Demographic descriptions did not vary greatly between participants who redeemed a voucher compared to those who did not. However, food voucher redemption was affected by discharge time: participants discharged during market operating hours had a higher proportion of voucher redemption 66, 41.5%) compared to participants discharged when the market was closed (20, 30.8%) (Appendix 1).

## DISCUSSION

In this prospective cohort study of adult patients seen at a county ED, a high proportion of respondents screened positive for food insecurity and accepted food vouchers; however, the redemption rate of food vouchers at the hospital’s co-located food market was low and often occurred greater than one week after voucher distribution.

The observed proportion of participants screening positive for food insecurity in this study (61%) is greater than in prior studies of ED patients, which historically ranged from 16–51%.^6^ ^,^ ^9^ ^,^ ^15^ ^,^ ^18^ ^–^ ^21^ Even when accounting for the effect of the COVID-19 pandemic, the observed proportion of participants screening positive for food insecurity in this study was higher than other studies during this period, including an identical screening process implemented in outpatient clinics at the study site (30–37%).^4^ ^,^ ^16^ Participant acceptance of a voucher was also higher than the 65% acceptance rate in Aylmard’s 2021 study and Bottino et al’s 2017 study acceptance rate of 17%.^18^ ^,^ ^22^ However, participant use of the food voucher resource was similar to prior acceptance rates for social services referrals and shows similar utilization of summer food programs that addressed food insecurity at a children’s ED.^18^ ^,^ ^23^

This study builds upon the work of Jahnes et al (2020), which addressed a similar problem through a different approach: the authors implemented a program in which patients were given a bag of food at the time of discharge without examination of eligibility criteria or further documentation. Bags included shelf-stable food as well as “no-cook bags” for individuals without cooking infrastructure. The first notable difference in results is that 3,000 food bags were distributed by Jahnes et al as opposed to 226 food vouchers in this study. While the monetary value of each food bag in Jahnes et al is not known, even if one assumed a cost of $10 per bag, more food was distributed in an operational program focused on food distribution rather than in this research study. The second notable difference was that this study’s approach allowed participants to choose what options best served their needs, including the purchase of other cooking items (eg, cooking spray or utensils), if those were needed more than food items. Additionally, the study protocol allowed distribution of perishable food, which has rarely been offered in similar ED-based programs. These differences highlight key tradeoffs between two different approaches: the ease of pre-made, ED-distributed food bags vs the customizability of a patients shopping for themselves. Future work should further characterize patient preferences between these strategies to provide critical insights into the circumstances in which one is preferred over the other by patients with social needs.

It was unexpected that despite high acceptance rates of food vouchers, less than half of all participants redeemed a voucher at the co-located hospital food market. A conceptual explanation of this discrepancy could be social risk vs social need; while the screening identified a social risk (ie, an adverse social condition associated with poor health) and provided resources directed at reducing a social risk, participants may not have perceived food as a social need (ie, an adverse social determinant of health for which they would have liked assistance and viewed as a priority).^24^ However, this is considered less likely due to the median redemption time of food vouchers of nine days, suggesting that using the voucher was important enough to return for redemption. A pragmatic explanation of the observed discrepancy is that the food market was initially designed for a food insecurity screening and intervention in the outpatient primary care setting (ie, weekdays, daytime hours), rather than the ED setting (ie, all days and hours). The misalignment between ED screening times and market hours appears to have modified the effect of food voucher distribution on redemption rates. The overall redemption rate was 38.9%; redemption rate for individuals discharged during market hours was 41.5%, while the redemption rate for individuals discharged after market hours was 30.8%). These results should prompt further consideration of unique aspects of ED operations when designing future food insecurity interventions within a hospital system.

It was also surprising that nearly 60% of participants experiencing food insecurity had an established primary care doctor prior to their ED evaluation. This finding contrasts with Robinson et al’s 2018 study, which found food insecurity was associated with lack of primary care.^3^ While this study was not designed to determine whether previous primary care appointments had screened for or addressed food insecurity, participant willingness to disclose a social risk during the ED encounter aligns with Cullen et al’s 2019 study, which found that families were more comfortable with social determinants screening in the ED rather than the primary care setting and supports ongoing efforts to screen for social needs in the ED.^25^ The observed follow-up rate of less than 50% and time to primary care appointment exceeding one month are consistent with prior observations by Loo et al (2013), Wallace et al (2021), and Zu et al (2006).^11^ ^,^ ^12^ ^,^ ^19^

These findings also highlight the important role of the ED in addressing social risks that are identified during ED screening. If screened risks are not addressed (and instead referred to outpatient physicians), follow-up may not occur for up to one month. One conceptual question that remains unanswered is what services (eg, primary care, social work, case management, nutrition/dietetics, community agencies, or multidisciplinary teams either in person or virtually) are most appropriate to refer patients to after they screen positive for a social need, such as food insecurity, in the ED. The study protocol opted to refer participants back to their primary care physician because food insecurity would likely require a more comprehensive social needs assessment, but researchers in future studies may wish to consider alternative strategies to address this question.

## LIMITATIONS

The study design was at risk of selection bias, participation bias, and contamination bias. Selection bias occurred during inclusion/exclusion wherein individuals arriving outside the study hours, individuals with psychiatric illness, patients presenting in extremis, minors, and individuals who could not provide written consent in English were excluded. Although subsequent quality improvement projects have addressed these populations, the research results presented here are not generalizable to patients outside the study population. Participation bias may have occurred due to the ethics requirement to disclose the risks and benefits of study participation, including a food voucher with monetary value; this may also explain the higher-than-expected proportion of participants screening positive for food insecurity among the study population. Additionally, participation bias may have occurred using a written screening tool that may have made individuals with low literacy less likely to participate, even though the RA protocol included offering that the screening questions be read aloud.

The single-center study design without longitudinal contact with study participants limits our ability to comment on contamination bias; it is possible that patients obtained connection to care from external resources rather than the study’s voucher program and printed resources and did not use the provided voucher for this reason. The low redemption rate of food vouchers was unexpected, and the study design and informed consent did not allow further investigation into the reasons for the low proportion of voucher redemption; thus, conclusions about the causes of this finding are limited. Finally, the unique aspects of project funding and market location limit this study’s generalizability to similar health systems with similar available resources.

## CONCLUSION

A high proportion of study participants screened positive for food insecurity and accepted a food voucher for a co-located resource addressing this social risk; however, voucher redemption rates were low and occurred greater than one week following distribution. These results imply that food insecurity screening and voucher distribution are feasible, but that co-location of resources alone may not completely address the social risk and should prompt consideration of resource accessibility (both location and hours), customizability, and patient preferences in treating social needs identified in the emergency department.

## Supplementary Information




