# Supplementary material for: Emergency Department Food Insecurity Screening, Food Voucher Distribution and Utilization: A Prospective Cohort Study
Source: West J Emerg Med. 2024 Sep 19;25(6):993–9. doi: 10.5811/westjem.18513 (PMC11610723; doi:10.5811/westjem.18513)
Supplement: Supplementary file 1 [file wjem-25-993-s001.docx]

**Appendix 1.** Demographic description of enrolled participants who screened positive for food insecurity and received a voucher, stratified by voucher utilization (n=224).

| Variable | Food Voucher Redeemed (n=86) | Voucher Not Redeemed (n= 138) |
| --- | --- | --- |
| Gender, *n (column %)* |  |  |
| Male | 41 (47.7) | 64 (46.4) |
| Female | 45 (52.3) | 74 (53.6) |
| Age, *median (IQR)* | 51 (36-58) | 43 (30-57) |
| Race, *n (column %)* |  |  |
| Black or African American | 60 (69.8) | 88 (63.8) |
| Native Hawaiian/Pacific Islander | 0 | 1 (0.7) |
| White | 26 (30.2) | 49 (35.5) |
| Ethnicity, *n (column %)* |  |  |
| Hispanic or Latino/a | 3 (3.5) | 16 (11.6) |
| Non-Hispanic | 83 (96.5) | 122 (88.4) |
| Emergency Department Discharge Time, *n (column %)* | |  |
| During Market Hours | 66 (76.7) | 93 (67.4) |
| After Market Hours | 20 (23.3) | 45 (32.6) |
